# EXecutive-Functions Innovative Tool (EXIT 360°): A Usability and User Experience Study of an Original 360°-Based Assessment Instrument

**DOI:** 10.3390/s21175867

**Published:** 2021-08-31

**Authors:** Francesca Borgnis, Francesca Baglio, Elisa Pedroli, Federica Rossetto, Sara Isernia, Lidia Uccellatore, Giuseppe Riva, Pietro Cipresso

**Affiliations:** 1 IRCCS Fondazione Don Carlo Gnocchi ONLUS, 20148 Milan, Italy; fborgnis@dongnocchi.it (F.B.); fbaglio@dongnocchi.it (F.B.); frossetto@dongnocchi.it (F.R.); sisernia@dongnocchi.it (S.I.); 2 Department of Psychology, Università Cattolica del Sacro Cuore, 20123 Milan, Italy; lidia.uccellatore01@icatt.it (L.U.); giuseppe.riva@unicatt.it (G.R.); 3 Applied Technology for Neuro-Psychology Lab., Istituto Auxologico Italiano, Istituto di Ricovero e Cura a Carattere Scientifico, 20149 Milan, Italy; e.pedroli@auxologico.it; 4 Faculty of Psychology, eCampus University, 22060 Novedrate, Italy; 5 Department of Psychology, Università degli Studi di Torino, 10124 Turin, Italy

**Keywords:** executive function, 360° environment, assessment, virtual reality, usability, user experience, engagement

## Abstract

Over the last few decades, several studies have shown the feasibility, acceptability, and efficacy of VR-based instruments in the early evaluation of executive dysfunction (ED) in psychiatric and neurologic conditions. Due to the negative impact of ED in everyday functioning, identifying innovative strategies for evaluating ED allows clinicians to detect executive impairment early and minimize its effects. This work aimed to test the usability and user experience (UX) of EXecutive-functions Innovative Tool 360° (EXIT 360°), a 360°-based tool for assessing ED. Seventy-six healthy subjects underwent an evaluation that involved (1) usability assessment using System Usability Scale and (2) evaluation of UX using the ICT-Sense of Presence and UX Questionnaire. Results showed a satisfactory level of usability (mean = 75.9 ± 12.8), with good scores for usability and learnability. As regards UX, EXIT 360° showed an absence of negative effects (mean = 1.79 ± 0.95) and high scores in ecological validity (mean = 4.32 ± 0.54) and engagement (mean = 3.76 ± 0.56). Moreover, it obtained good scores in efficiency (mean = 1.84 ± 0.84), originality (mean = 2.49 ± 0.71), and attractiveness (mean = 1.93 ± 0.98). Interestingly, demographic characteristics and technological expertise had no impact on the performance (*p* > 0.05). Overall, EXIT 360° appeared to be a usable, learn-to-use, engaging, and creative tool with irrelevant negative effects. Further studies will be conducted to evaluate these aspects in the clinical population.

## 1. Introduction

Over the last few decades, virtual reality (VR) appeared to be a promising tool for assessing executive functions (EFs) (Borgnis et al., submitted), a complex construct that involves several higher-order cognitive and behavioral skills with a key role in daily life and independent functioning [1,2]. Several studies have shown the feasibility, acceptability, and efficacy of VR-based instruments in the early evaluation of executive dysfunction in both psychiatric (e.g., schizophrenia) [3] and neurologic (acute, neurodegenerative, and neurodevelopment) conditions [4,5,6]. Executive dysfunction represents, in these clinical conditions, a significant health challenge due to its high negative impact on daily functioning (i.e., preparing meals, managing money, shopping, school, and work performances) [7]. VR-based tools allow clinicians to identify novel strategies for evaluating EFs in real-life scenarios to detect executive impairment early and minimize its effects, improving everyday functioning [8]. Specifically, VR-based instruments, such as 360° VR video (in which the 360° technologies were used to provide a VR experience), allow to carry out different tasks in ecologically valid and controlled environments [9,10,11,12]. Thus, clinicians could observe their patients in an environment with constraints similar to those found in real life, directly monitoring the executive difficulties that influence everyday life and independent functioning. The tasks involved complex real life situations (e.g., shopping and cooking) that required subjects to use several EFs [13], mirroring the cognitive demands of daily functioning [14]. Moreover, VR-based tools are highly flexible and programmable instruments that guarantee the controlled and precise presentation of a large variety of stimuli [9] and distractions/stressors that patients may meet in their everyday life.

In this framework, we developed EXecutive-functions Innovative Tool 360° (EXIT 360°), an original 360°-based instrument for a multicomponent, ecologically valid evaluation of executive functioning in the neurologic population [15]. EXIT 360° consists of a new task for EFs delivered via a comfortable mobile-powered VR headset. EXIT 360° enables subjects to engage in a “game for health” delivered via smartphones, where they have to perform different everyday subtasks in 360° domestic environments (e.g., kitchen, bedroom). EXIT 360° allows an immersive VR experience by 360° spherical imageries depicting a real ecological environment.

In EXIT 360°, the participants have the main goal to go out of the domestic path in the shortest possible time. To do this, participants have to plan a strategy and overcome seven different steps (subtasks) of increasing complexity, designed to evaluate several components of executive functioning simultaneously and quickly [15].

As widely demonstrated [16,17,18], it is necessary to consider some key elements in using technological tools, such as VR-based instruments. Specifically, several studies have shown the crucial role of assessing usability, user experience, and engagement in developing VR-based tools [18,19]. Recently, Sauer and colleagues proposed an innovative higher-level concept, the “interaction experience” expected to provide several benefits to users which involved both usability and user experience [17]. The usability assessment allows understanding the “degree to which a subject is able to use a system to achieve specific goals effectively, efficiently, and satisfactorily within a well-defined context of use” [20]. Specifically, the usability consists of three main factors, connected to the characteristics and the goals of the users and the context of use: the effectiveness (i.e., possibility for the users to achieve goals), efficiency (i.e., users’ efforts to reach the aim), and satisfaction (i.e., users’ thoughts about their interaction with the system). Overall, the usability evaluation allows for understanding any difficulties that could affect subjects’ performance.

As regards the user experience, it is defined as “a person’s perceptions and responses that result from the use or anticipated use of a product, system or service” [21]. Over the years, researchers have also paid attention to improving user experience in virtual environments by working on several domains: sense of presence, sense of realism, engagement, enjoyment, and side effects in the development of digital contents [3,22]. For example, previous evidence showed sickness during virtual environments such as dizziness, headache, sweating, and nausea [9]. The feeling of cybersickness can lead to unpleasant experiences for the users, affecting their performance and significantly decreasing the test results’ validity. Another important aspect of evaluating concerns the level of familiarity of users with technology, above all older adults, since poor performance in the test could be due to insufficient knowledge of how VR works [23].

Finally, the developers have tried to increase engagement in virtual environments by improving the ecological validity of VR-based tools. Specifically, the VR-based instruments want to offer contexts similar to the real world, allowing patients to live a first-person experience “like in real-life”. Focusing on enjoyment and attractiveness enables the increase of users’ motivation and participation and the decrease of anxiety typical of neuropsychological evaluation.

In this study, we evaluated if EXIT 360° was able to meet all these significant characteristics: good usability, absence of side effects, high sense of presence, ecological validity, enjoyment, and attractiveness.

## 2. Materials and Methods

### 2.1. Participants

Seventy-six healthy subjects were consecutively recruited at IRCCS Fondazione Don Carlo Gnocchi in Milan, according to the following inclusion criteria: (a) age between 18 and 90 years; (b) education ≥5; (c) absence of cognitive impairment as determined by the Montreal Cognitive Assessment test [24] (MoCA score ≥17.54, cut-off of normality), corrected for age and years of education according to Italian normative data [25]; and (d) ability to provide written, signed informed consent. Exclusion criteria for all subjects were: (a) hearing or visual impairment that could have compromised the assessment with EXIT 360°; (b) systemic, psychiatric, or neurological illnesses; and (c) overt visual hallucinations or vertigo.

The study was approved by the “Fondazione Don Carlo Gnocchi ONLUS” Ethics Committee in Milan. All participants were provided with a complete explanation of the purpose and risk of the study before the preparation of the consent form and written informed consent was obtained based on the revised Declaration of Helsinki (2013).

### 2.2. Procedure

The study consisted of a one-session evaluation that involved three main phases: a pre-task evaluation, followed by the EXIT 360° session, and a post-task evaluation [26].

#### 2.2.1. Pre-Task Evaluation

After signing written informed consent, participants underwent an evaluation of their global cognitive profile through the Montreal Cognitive Assessment (MoCA) test, a sensitive screening tool to exclude the presence of cognitive impairment.

After that, the psychologist collected participants’ socio-demographic data (e.g., age, gender, education level) and technological expertise. Specifically, the participants evaluated their perceived level of familiarity and competence with several technologies: tablet, smartphone, computer, and Internet (including social network). To test their level of familiarity with technologies, the psychologist administered a 5-point scale (from “never” to “every day”) that evaluated “how often, in the last year, did you use…”; while the competence scale involved a 5-point scale (from “nothing” to “much”) to investigate “how competent do you feel in using…”.

#### 2.2.2. EXIT 360° Session

After the preliminary screening, all participants underwent an evaluation with EXIT 360°. The psychologist started the administration by inviting participants to sit on a swivel chair and wear the Head Display Mobile (HDM). Before wearing the HDM, the psychologist provided the following general instruction of the task:

“*You will now wear a headset. Inside this viewer, you will see some 360° rooms of a house. To visualize the whole environment, I ask you to turn on yourself; you are sitting on a swivel chair for this reason. Within these environments, you will be asked to perform some tasks*”.

In the case of presbyopia, participants could wear their glasses.

EXIT 360° was developed using the InstaVR^©^ software, which allowed for organizing virtual environments and several tasks in a single experience. Specifically, the tool consists of 360° immersive domestic photos as virtual environments in which participants have to perform seven subtasks of increasing complexity: (1) Let’s Start; (2) Unlock the Door; (3) Choose the Person; (4) Turn On the Light; (5) Where Are the Objects?; (6) Solve the Rebus; and (7) Create the Sequence (for a detailed description, see [15]). Briefly, the subtasks reproduce common scenarios of everyday life that ask the subject to solve specific assignments according to the instructions of each subtask. Specifically, in the first task, participants have to observe a map and choose the path that allows them to complete getting to the exit in the shortest possible time. In “Unlock the Door”, the subjects have to open a door choosing the three options. The task “Choose the Person” requires participant to explore the room and select the correct person according to a specific instruction. In task 4, the subjects are immersed in a completely dark room and they have to choose a correct object to continue the journey. In task 5, participants have to identify the piece of furniture (among several furniture) on which four specific objects are placed. In the following task, participants have to complete a rebus. Finally, in the last task, subjects have to memorize a sequence of numbers and report them in reverse.

Overall, all these tasks are designed to evaluate different components of executive functioning (multicomponent assessment) simultaneously and quickly (Table 1). The level of complexity of the tasks changes according to the cognitive load and the presence of confounding variables.

To respond to these requests, the subject has to choose between three or more “options”, the one that will allow him to solve the assignment in the best possible way. In the HDM, they see a small white dot/square, a “pointer” that follows their gaze; therefore, when subjects want to answer within the environment, they have just to move their head and position the white dot over the answer for a few seconds and the answer will be selected automatically. In this way, it is possible to collect participants’ answers without learning to use complex tools (e.g., joysticks). Interestingly, to provide the instructions in a standardized way, the instructions received from the participants were previously recorded and inserted within the virtual environments.

EXIT 360° evaluation involved a preliminary familiarization phase to familiarize the subjects with the technology and control potential adverse effects (e.g., dizziness, nausea). Participants were completely immersed in a neutral 360° environment representing a living room, including a table with a plant, a sofa, various chairs, and objects spread throughout the room. The examiner asked participants to explore the environments freely, saying what they saw in the room. Upon completing the familiarization phase, participants were asked to indicate the possible presence of nausea or malaise. If side effects occurred, the examiner had to stop the test immediately.

In contrast, subjects were immersed in 360° environments that represented a living room. The experimental session (and time registration) began when the participants heard the following instruction: “*You are about to enter a house. Your goal is to get out of this house in the shortest time possible. To exit, you will have to complete a path and a series of tasks that you will encounter along your way. Are you ready to start*?”. During their evaluation, the subjects had to explore the 360° environment freely, simply through the movement of the head as it happens in real situations [27]. As previously said, participants had to perform seven subtasks. EXIT 360° recorded all the subjects’ responses and all participants completed the seven subtasks: if the subjects chose a wrong alternative, they received only one point (vs. two points for a correct answer). For this reason, we evaluated the usability and user experience on the whole task.

##### Outcome Measures

The following indices were calculated:

1. Correct answer for each subtask and total score;
2. Sub-task reaction time: the time (in seconds) from the start of a sub-task until the participant provided an answer; and
3. Total reaction time: the time in seconds registered from the first instruction until the participant provided the last correct answer.

#### 2.2.3. Post-Task Evaluation

At the end of the EXIT 360° task evaluation, subjects had to rate:

1. The usability of the technological instrument by the **System Usability Scale** (SUS) [28], a short questionnaire composed of ten items (five with a positive value and five with a negative value) on a 5-point scale, from “completely disagree” to “strongly agree”. The score ranges from 0 to 100 and indicates the overall usability of the system. SUS is a valid, reliable, and quick-to-use scale widely used to evaluate the usability of a wide range of technological devices. Furthermore, the scale takes into account two main aspects that affect the user experience: usability, which indicates the ease with which the user uses the system (scores 1–4), and learnability, which represents the ease with which the user learns to use the system (scores 1–4) [29].
2. The user experience through:
  a. three items of the **Flow Short Scale** (5-point scale: from low to high) to assess their perceived level of abilities in coping with the task, of challenges, and the perceived challenge-skill balance [30].
  b. four items (5-point scale: from low to high) from the subscale enjoyment of the **Intrinsic Motivation Inventory** (IMI) [31] to evaluate participants’ appreciation of the proposed activity (i.e., boring, pleasant, fun and activating). The item boring scores were reversed to align it with the remaining items; therefore, in the whole scale, a low value in the items reflects a negative perception of the experience with EXIT 360°.
  c. forty-four items (5-point scale: from 1: “strongly disagree” to 5: “strongly agree”) from the scale **Sense of Presence Inventory** (ICT-SOPI) to evaluate user’s engagement. Precisely, this questionnaire consists of 44 items divided into four subgroups (each subgroup is generated by calculating a mean of all completed items contributing to each factor): (1) “spatial physical presence”: feeling of being in a physical space in the virtual environment and having control over it (19 items, e.g., “I felt I could interact with the environment shown”); (2) engagement: the tendency to feel psychologically and pleasantly involved in the virtual environment (13 items, e.g., “I was sorry my experience was over”); (3) ecological validity: the tendency to perceive the virtual environment as real (6 items, e.g., “The environment shown seemed natural to me”); and (4) negative effects: adverse psychological reactions (6 items, e.g., “I felt nauseous”). Moreover, ICT-SOPI is divided into two parts: thoughts and feelings after experiencing the environment (Part A) or while the user was experiencing the environment (Part B) [32].
  d. **User Experience Questionnaire**, a 26-item scale (semantic differential scale: each item consists of two opposite adjectives, e.g., boring vs. exciting) that allows calculating six different domains: (1) attractiveness (overall impression of the product), (2) perspicuity: easily to learn how to use the product; (3) efficiency (user’s effort to solve tasks); (4) dependability (feeling of control of the interaction); (5) stimulation (motivation to use the product); and (6) novelty: (innovation and creation of product) [33,34,35].

### 2.3. Statistical Analysis

Descriptive statistics included frequencies, percentages, and median and interquartile range (IQR) for categorical variables and mean and standard deviation (SD) for continuous measures. The normality of data distribution was assessed using the Kolmogorov–Smirnov test. Pearson’s correlation was applied to compare the scores of usability, user experience, and technological experience. Moreover, a one-way ANOVA non-parametric (Kruskal–Wallis) and Chi-square were calculated to verify possible differences in education and gender in age group. Furthermore, a one-way ANOVA between subjects (post-hoc: Bonferroni test) was conducted to evaluate any significant differences in technological expertise due to age group. Finally, ANCOVA between subjects (covariate: education) was performed to assess possible differences in usability score due to age. All statistical analyses were conducted using Jamovi 1.6.7 software. A statistical threshold of *p* < 0.05 was considered statistically significant.

## 3. Results

### 3.1. Participants

Table 2 reports the demographic and clinical characteristics of the whole sample. The subjects (*n* = 76) were predominantly female (M:F = 28:48) with a mean age of 53.5 years (SD = 20.30, range = 20–89) and age of education nearly 13 (IQR = 13–18, range 5–18). All participants included in the study showed an absence of cognitive impairment (MoCA correct score = 25.9 ± 2.63).

Table 3 reports the scores of the demographic characteristics of participants divided for the age group. The comparison between the seven age groups showed a significant difference in the education (χ^2^ (6) = 29; *p* < 0.001), but not in the sex (χ^2^ (6) = 7.1; *p* = 0.312).

Specifically, results showed a significant difference between group 20–29 and 60–69 (W = −4.711; *p* < 0.05) and 70–79 (W = −4.711; *p* < 0.05). Moreover, a significant difference appeared between group 30–39 and 50–59 (W = −4.31; *p* < 0.05), 60–69 (W = −5.04; *p* < 0.05), and 70–79 (W = −4.82; *p* < 0.05).

### 3.2. Technological Expertise

The mean score of the ad hoc 5-point scale for evaluation of perceived level of familiarity with technologies was 3.41 ± 1.8 (i.e., participants used the technology about once a week). Specifically, 25% of subjects reported a low (<3) familiarity with technologies, with 28.9% that showed a good (≥4—at least two or three times a week) familiarity with several technologies. Moreover, the mean score of the ad hoc 5-point competence questionnaire was 3.48 ± 1.11, indicating a score between “little” and “enough”. In particular, 28.9% of participants showed a low (<3) competence with technologies, with 30.03% showing a good (≥4—enough or much) competence with several technologies. Analyzing the possible difference between age groups in levels of competence and familiarity with technologies, data showed a significant difference between many age groups both in familiarity (F (6,69) = 12.6; *p* < *0*.001) and competence (F (6,69) = 22.2; *p* < *0*.001). Specifically, results showed a significant difference in familiarity between group 80–89 and all other age groups, except for 70–79 (*p* > *0*.05). Moreover, a significant difference appeared between groups 70–79 and 20–29 (*p* < 0.001) and 30–39 (*p* < 0.001). Regarding the level of competence, results indicated a significant difference between group 80–89 and all other age groups (*p* < *0*.05). Moreover, a significant difference appeared between group 70–79 and respectively 20–29 (*p* < 0.001), 30–39 (*p* < 0.001), and 40–49 (*p* < *0*.05). In addition, group 60–69 showed a significant difference with 20–29 (*p* < *0*.001) and 30–39 (*p* < *0*.05) groups. Finally, group 50–59 showed a difference only with 20–29 (*p* < *0*.05).

### 3.3. Usability

**Figure 1:**
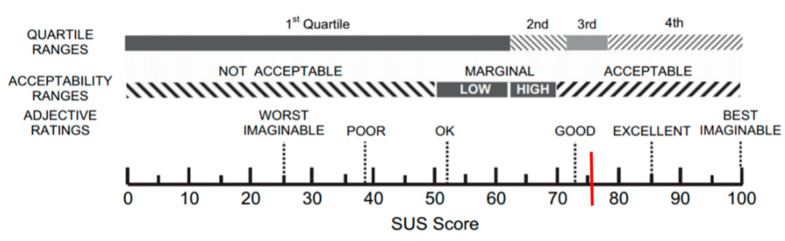
A graphic representation of the SUS score. The figure is modified from the original version (Bangor et al. 2008).

The mean value of the usability, calculated with the SUS, was 75.9 ± 12.8, indicating a satisfactory level of usability, according to the scale’s score acceptability ranges (cut off = 68) and adjective ratings (Figure 1).

**Figure 2:**
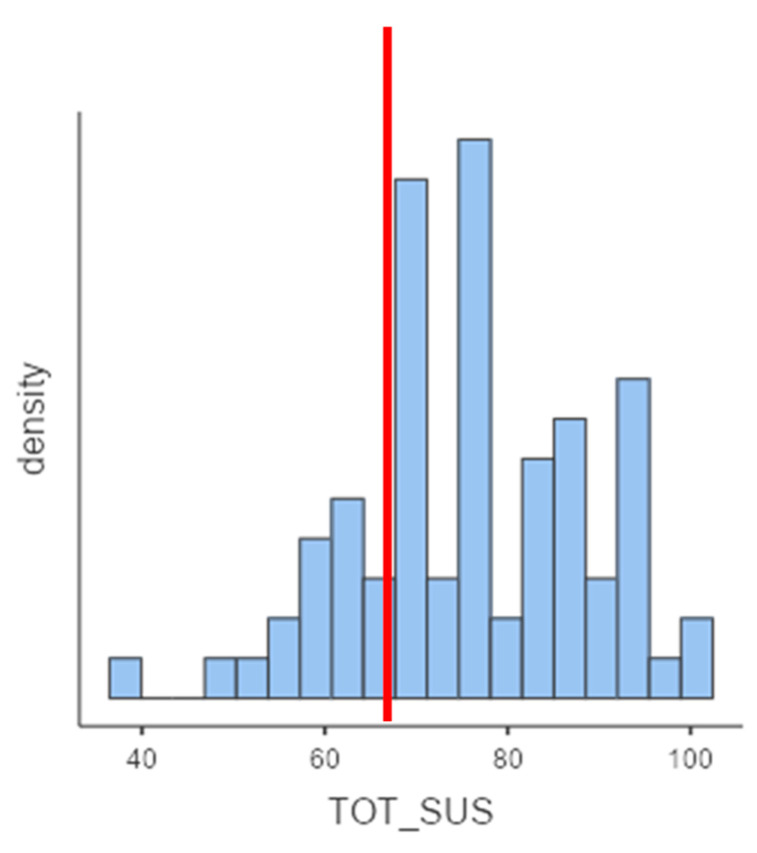
A graphic representation of SUS score’s distribution. The red line indicates cut-off of the SUS scale.

Specifically, according to the cut-off score (cut-off = 68), more than 77% of participants showed scores above the cut-off (Figure 2).

**Figure 3:**
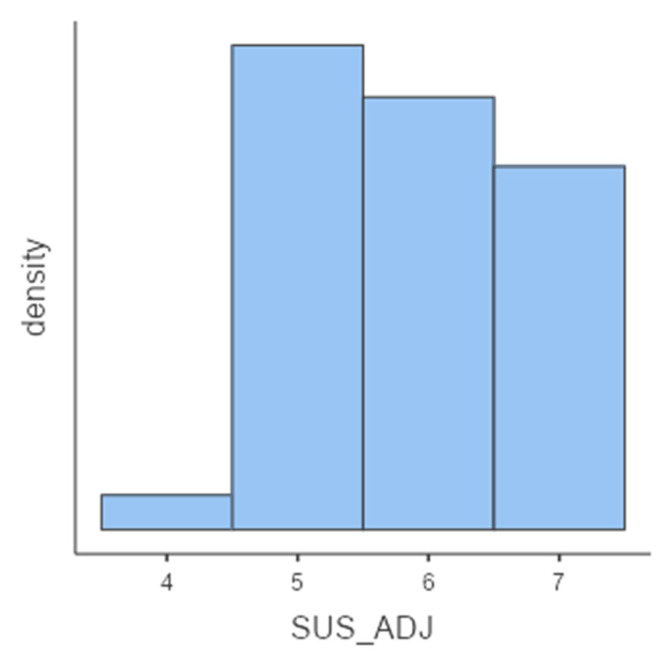
Graphic representation of the adjective rating.

In addition, according to the adjective rating, 36.8% of subjects evaluated EXIT 360° as “good”, 32.9% as “excellent”, and 27.6% “best imaginable” [36] (Figure 3).

Interestingly, participants provided good and promising scores to two main aspects that affect the user experience: usability (mean = 3.07 ± 0.55) and learnability (mean = 2.91 ± 0.738). Specifically, 14.5% and 15.8% of participants showed low scores (<2.5) respectively at usability and learnability.

### 3.4. User Experience

The three items of the Flow Short Scale showed a high score in the perceived level of skill in performing EXIT 360° (median = 5, IQR = 4–5) and allowed for evaluating the level of challenge of EXIT 360°, also in based on own abilities, such as balance/appropriate (median = 3; IQR = 3).

**Figure 4:**
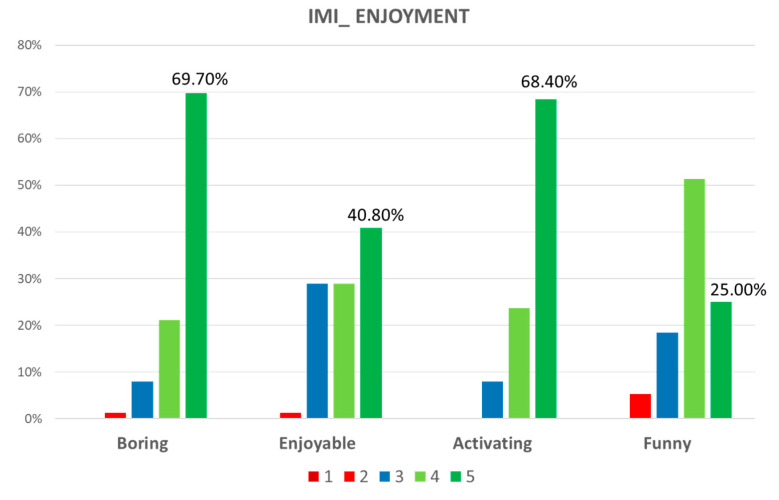
Graphic representation of the domains of the Intrinsic Motivation Inventory.

Figure 4 showed that the subscale enjoyment of the IMI obtained high scores (≥4) in all items: boring (median = 5, IQR = 4–5), enjoyable (median = 4, IQR = 3–4), activating (median = 5, IQR = 4–5), and funny (median = 4, IQR = 4–4.25).

**Figure 5:**
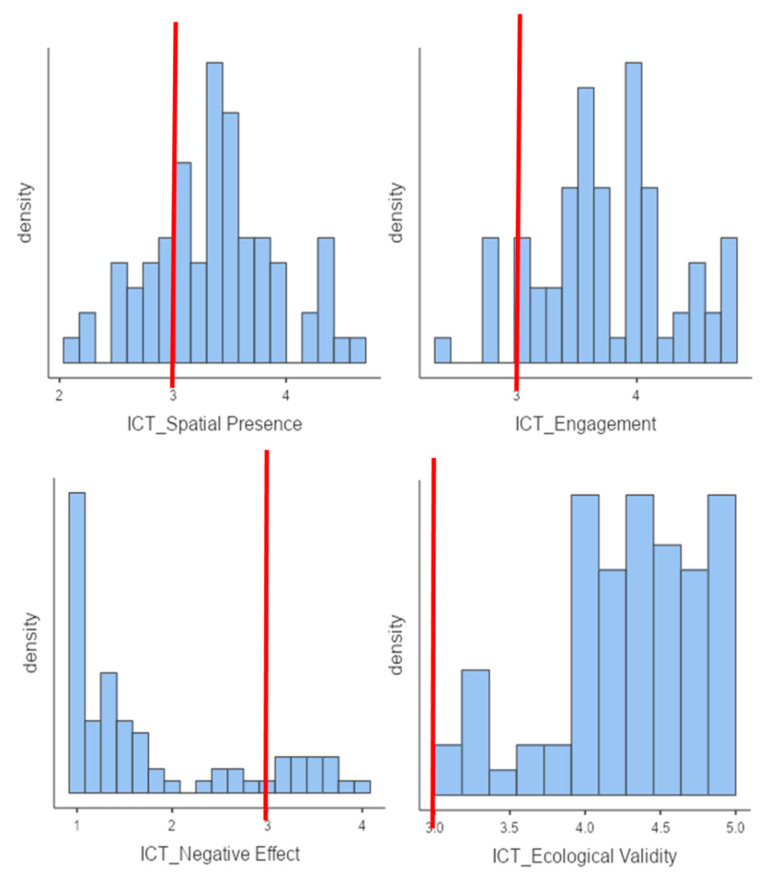
Graphic representation of five ICT-SOPI dimensions. The red lines indicate a neutral score.

Figure 5 showed good scores in all ICT-SOPI dimensions: spatial presence (mean = 3.38 ± 0.55, range = 2.11–4.63), engagement (mean = 3.76 ± 0.56, range = 2.38–4.77), ecological validity (mean = 4.32 ± 0.54, range = 3–5), and negative effects (mean = 1.79 ± 0.95, range = 1–4). Specifically, 10.4% (*n* = 11) of participants reported the presence of negative effects (e.g., nausea, vertigo), but only one subject showed relevant side effects. Moreover, 75% showed good scores of spatial presence (vs. 9.2% that showed scores ≤ 2.5—e.g., (“I felt I could interact with the environment shown”) and 89.5% indicated a good level of engagement while performing EXIT 360° (e.g., “I would have liked the experience to continue”). Finally, 97.4% of participants gave EXIT 360° a good score in terms of ecological validity (“I had the feeling that the environment shown was part of the real world”), with more than 84% that showed high scores (≥4).

**Figure 6:**
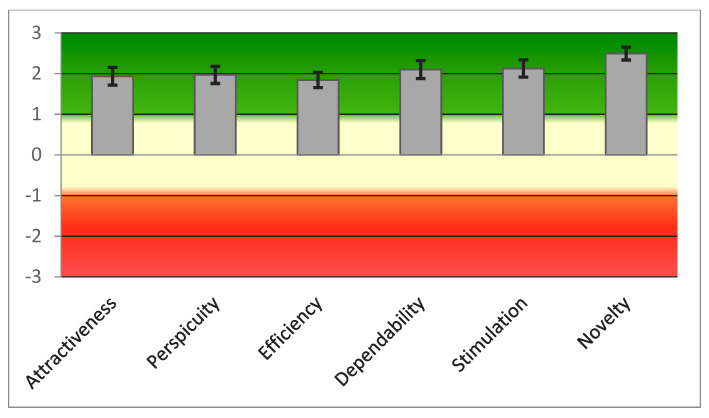
Graphic representation of scores of the six UEQ scales.

The UEQ questionnaire showed positive evaluation (>0.8) in all 26 items. Figure 6 showed good scores in all scales according to the questionnaire’s score ranges (range between −3, horribly bad, and +3, extremely good).

Table 4 shows in detail the mean scores of all scales with their respective high values of internal consistency (Alpha-coefficient > 0.7) [37].

Moreover, the UEQ’s scales can be grouped into pragmatic quality (involving perspicuity, efficiency, dependability) and hedonic quality (non-task-related quality aspects—stimulation and originality). Specifically, EXIT 360° obtained a good score in pragmatic quality (mean = 1.97) and a high score in hedonic quality (mean = 2.31).

**Figure 7:**
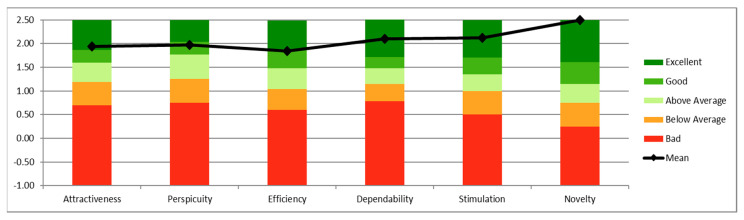
Comparison between means of each UEQ scales and values from a benchmark data set.

Finally, the means of each UEQ scale were compared to existing values from a benchmark data set (containing data from 20,190 persons from 452 studies) [35]. Results showed that the scales attractiveness, dependability, stimulation, and novelty obtained excellent evaluation, belonging to the range of the 10% best results (Figure 7).

### 3.5. Correlation

**Figure 8:**
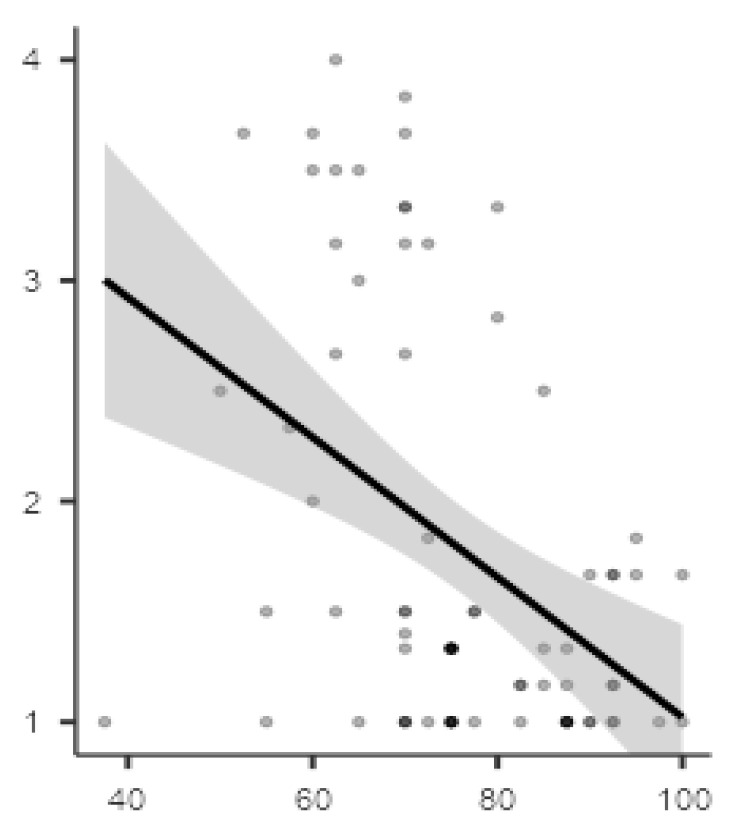
Graphic representation of negative correlation between negative effects and SUS total score.

Pearson’s correlation showed the absence of significant linear correlation between the SUS total score and the demographic characteristics, particularly age (r = −0.078, *p* = 0.503) and education (r = −0.107; *p* = 0.356). Moreover, the ANCOVA-between subjects (covariate: education) showed no significant differences of age group in SUS total score (F (6,68) = 1.02; *p* = *0*.419). Interestingly, this analysis also showed the absence of impact of education in the SUS total score (*p* = *0*.159). Furthermore, Pearson’s correlation has underlined the absence of significant correlation between SUS total score and technological expertise measured by the two ad hoc questionnaires of familiarity (r = 0.012; *p* = 0.915) and competence (r = 0.177; *p* = 0.127). In contrast, Pearson’s correlation showed significant and positive linear correlation between SUS total score and three ICT-SOPI domains, spatial presence (r = 0.247; *p* < 0.05), engagement (r = 0.495; *p* < 0.001), and ecological validity (r = 0.466; *p* < *0*.001). Finally, Figure 8 highlights the presence of a significant and negative correlation between the SUS total score and the ICT-SOPI domain negative effect (r = −0.43; *p* < 0.001).

## 4. Discussion

In recent years, there has been a growing interest in using 360° VR video for an ecologically valid assessment of executive functioning in the neurologic population [11,12]. In view of the relevant impact of executive dysfunction on everyday life [7], the identification of innovative strategies for evaluating EFs in real-life scenarios allows a promising solution for detecting these executive impairments and minimizing their effects, improving everyday functioning [8]. In this framework, EXIT 360° fits perfectly into the ongoing transformation of traditional neuropsychological assessment [9,10,38]. EXIT 360° aimed to be an original 360°-based instrument for a multicomponent, ecologically valid evaluation of executive functioning in the neurologic population [15]. This work was borne by the need, amply demonstrated, to consider usability, user experience, and engagement as key elements in developing and using technological tools [16,17,18].

Our work involved seventy-six healthy participants, predominantly female with a mean age of 53.5 years. At the pre-task evaluation, all subjects showed an absence of cognitive impairment according to inclusion criteria. Participants were able to successfully carry out EXIT 360° without relevant adverse effects, as demonstrated by previous studies with 360°-based instruments [11,12].

As regards usability evaluation, data showed a good usability score (mean = 75.9 ± 12.8), evaluated by SUS, indicating a satisfactory level of usability, according to the scale’s score acceptability ranges and adjective ratings [28]. Specifically, 36.8% of subjects considered EXIT 360° good, 32.9% as excellent, and 27.6% best imaginable. Moreover, participants provided good and interesting scores for the variables of usability and learnability, indicating that EXIT 360° can be considered an easy-to-use technological tool and an instrument easy to learn [29]. This interesting usability result allows us to conclude that EXIT 360° showed high effectiveness (i.e., possibility for the users to achieve goals), efficiency (i.e., users’ efforts to reach the aim), and satisfaction (i.e., users’ thoughts about their interaction with the system) [20]. Therefore, it is possible to support that any subjects’ low performance does not depend on technological problems. Interestingly, this result was not influenced by either demographic characteristics (age and education) and technological expertise measured by the two ad hoc questionnaires of familiarity and competence. Overall, according to these results, no adaptation of our system would be necessary.

As regards the user experience, EXIT 360° showed promising results in terms of absence of side effects, high sense of presence, ecological validity, enjoyment, and attractiveness. Firstly, participants showed high scores in the perceived level of skill in performing EXIT 360° and evaluated the subtasks of EXIT 360° as balanced/appropriate with respect to their abilities. Secondly, subjects supported a positive overall impression of the EXIT 360°, evaluating it as enjoyable, attractive, activating, funny, friendly, and not boring. Moreover, EXIT 360° demonstrated a good pragmatic quality as it appeared: (1) efficient, fast, and organized (efficiency); (2) understandable, easy to learn, and clear (perspicuity); and (3) supportive and secure (irrelevant side effects) (dependability). In addition, EXIT 360° showed excellent hedonic quality in terms of stimulation (exciting, interesting, and motivating) and novelty (creative, innovative). Interestingly, results showed that the scales attractiveness, dependability, stimulation, and novelty obtained excellent evaluation compared to existing values from benchmark data [39]. Finally, EXIT 360° appeared as an engaging and challenging tool with high spatial presence (“I felt I could interact with the environment shown”), good level of engagement while performing EXIT 360° (e.g., “I would have liked the experience to continue”), excellent ecological validity, and few adverse effects (only one subject reported the presence of relevant adverse effects such as nausea and vertigo). These interesting results appear to strongly agree with previous studies that highlighted the importance of sense of presence, sense of realism, engagement, enjoyment, and side effects in the development of digital contents [3,22].

Finally, data showed a relationship between usability score and user experience evaluation by ICT-SOPI domains spatial presence, engagement, and ecological validity (positive correlation) and negative effect (negative correlation). This conclusion supported the recent innovative higher-level concept of interaction experience, proposed by Sauer and colleagues, in which both usability and user experience appeared as key elements to provide more benefits in using technological tools [17].

Overall, EXIT 360° allowed an ecologically valid evaluation without relevant sicknesses such as dizziness, headache, sweating, and nausea that lead to unpleasant experiences for the users, affecting their performance and significantly decreasing the test results’ validity [9]. Moreover, our results showed that the level of familiarity of users with technology does not affect the performance in the test [23]. In addition, the high levels of enjoyment and attractiveness of EXIT 360° enabled the increase of users’ motivation and participation and the decrease of anxiety typical of neuropsychological evaluation.

### Limitations and Future Perspectives

The present work has some limitations. Firstly, the technological device used was entry-level: the 360° mobile-powered devices currently available on the market have much higher quality (e.g., Oculus Quest), which would allow for improving the quality of 360° images, providing a better and more realistic experience. Secondly, it is necessary to evaluate the usability, user experience, and engagement of EXIT 360° with the clinical population. Moreover, to date, EXIT 360° can be considered an initial prototype that needs further validation steps to become a valid and standardized instrument for assessing EFs. For this reason, it will be necessary to evaluate the convergent validity of EXIT 360°, comparing it with the traditional neuropsychological battery for executive functions (e.g., Frontal Assessment Battery) and its effectiveness in discriminating between healthy control subjects and patients with executive dysfunctions.

## 5. Conclusions

Results of the present study are very promising and interesting regarding usability, user experience, and engagement of EXIT 360°. Overall, participants obtained a positive global impression of the tool, evaluating it as usable, learn-to-use, clear, enjoyable, attractive, and friendly. Moreover, EXIT 360° is an efficient, fast, and organized instrument, with excellent hedonic quality in terms of stimulation (exciting and interesting) and originality. Finally, EXIT 360° also appeared to be an engaging and challenging tool with high spatial presence, excellent ecological validity, and irrelevant adverse effects. Interestingly, neither demographic characteristics (age and education) nor technological expertise influenced the encouraging results. Further studies will have to be conducted to also evaluate these aspects in a clinical population.

## Figures and Tables

**Table 1:** Subtasks and related executive functions.

	Planning	Decision-Making	Problem-Solving	Divided Attention	Visual Searching	Selective Attention	Reasoning	Working Memory
Task 1	x							
Task 2		x						
Task 3			x	x				
Task 4		x	x					
Task 5			x		x	x	x	
Task 6	x		x				x	
Task 7								x

**Table 2:** Demographic and clinical characteristics of the whole sample.

		Subjects (*n* = 76)
**Age** (years)	*Mean (SD)*	53.5 (20.30)
**Sex** (M:F)		28:48
**Age of education** (years)	*Median (IQR)*	13 (13–18)
**MoCA_raw score**	*Mean (SD)*	26.9 (2.39)
**MoCA_correct score**	*Mean (SD)*	25.9 (2.63)

M = male; F = female; SD = standard deviation; IQR = interquartile range; *n* = number; MoCA = Montreal Cognitive Assessment.

**Table 3:** Demographic characteristics of participants divided by the age group.

	20–29 (*n* = 13)	30–39 (*n* = 11)	40–49 (*n* = 10)	50–59 (*n* = 11)	60–69 (*n* = 11)	70–79 (*n* = 10)	80–89 (*n* = 10)	Group Comparison *p*-Value (*)
***n* (%)**	17.1%	14.5%	13.2%	14.5%	14.5%	13.2%	13.2%	
**Sex** (M:F)	4:9	3:8	6:4	2:9	4:7	6:4	3:7	0.312
**Education** **Median (IRQ)**	16 (16–18)	18 (17–18)	16.5 (13–18)	13 (13–13)	8 (8–13)	10.5 (8–13)	13 (5.75–16.8)	< 0.001 *

M = male; F = female; SD = standard deviation; IQR = interquartile range; *n* = number; (*) = significant difference.

**Table 4:** Scores of the six UEQ scales.

	Mean	SD	Confidence Interval		Alpha-Coefficient
**Attractiveness**	1.934	0.976	1.715	1.715	0.92
**Perspicuity**	1.967	0.934	1.757	1.757	0.87
**Efficiency**	1.842	0.840	1.653	1.653	0.79
**Dependability**	2.099	0.986	1.877	1.877	0.86
**Stimulation**	2.125	0.942	1.913	1.913	0.88
**Novelty**	2.493	0.705	2.335	2.335	0.90

SD = standard deviation.

## Data Availability

Data can be obtained upon reasonable request to the corresponding author.
